# Preparation of Pre-Confluent Retinal Cells Increases Graft Viability *In Vitro* and *In Vivo*: A Mouse Model

**DOI:** 10.1371/journal.pone.0021365

**Published:** 2011-06-29

**Authors:** Kevin P. Kennelly, Deborah M. Wallace, Toby M. Holmes, Deborah J. Hankey, Timothy S. Grant, Cliona O'Farrelly, David J. Keegan

**Affiliations:** 1 Catherine McAuley Clinical Research Centre, University College Dublin, Dublin, Ireland; 2 Department of Ophthalmology, Mater Misericordiae University Hospital, Dublin, Ireland; 3 Institute of Ophthalmology, University College London, London, United Kingdom; 4 School of Biochemistry and Immunology, Trinity College Dublin, Dublin, Ireland; 5 School of Public Health and Population Science, University College Dublin, Ireland; The University of Hong Kong, Hong Kong

## Abstract

**Purpose:**

Graft failure remains an obstacle to experimental subretinal cell transplantation. A key step is preparing a viable graft, as high levels of necrosis and apoptosis increase the risk of graft failure. Retinal grafts are commonly harvested from cell cultures. We termed the graft preparation procedure “transplant conditions” (TC). We hypothesized that culture conditions influenced graft viability, and investigated whether viability decreased following TC using a mouse retinal pigment epithelial (RPE) cell line, DH01.

**Methods:**

Cell viability was assessed by trypan blue exclusion. Levels of apoptosis and necrosis in vitro were determined by flow cytometry for annexin V and propidium iodide and Western blot analysis for the pro- and cleaved forms of caspases 3 and 7. Graft viability in vivo was established by terminal deoxynucleotidyl transferase dUTP nick end labeling (TUNEL) and cleaved caspase 3 immunolabeling of subretinal allografts.

**Results:**

Pre-confluent cultures had significantly less nonviable cells than post-confluent cultures (6.6%±0.8% vs. 13.1%±0.9%, p<0.01). Cell viability in either group was not altered significantly following TC. Caspases 3 and 7 were not altered by levels of confluence or following TC. Pre-confluent cultures had low levels of apoptosis/necrosis (5.6%±1.1%) that did not increase following TC (4.8%±0.5%). However, culturing beyond confluence led to progressively increasing levels of apoptosis and necrosis (up to 16.5%±0.9%). Allografts prepared from post-confluent cultures had significantly more TUNEL-positive cells 3 hours post-operatively than grafts of pre-confluent cells (12.7%±3.1% vs. 4.5%±1.4%, p<0.001). Subretinal grafts of post-confluent cells also had significantly higher rates of cleaved caspase 3 than pre-confluent grafts (20.2%±4.3% vs. 7.8%±1.8%, p<0.001).

**Conclusion:**

Pre-confluent cells should be used to maximize graft cell viability.

## Introduction

Retinal cell transplantation has been proposed as a therapeutic option for non-neovascular retinal degenerations[1]. This aims to replace atrophic or dysfunctional retina or retinal pigment epithelium (RPE) with healthy donor cells, including primary cell cultures, immortalized cell lines and stem cells. RPE grafts in the Royal College of Surgeons rat model of retinal degeneration have been shown to prevent overlying photoreceptor degeneration[2] and delay deterioration in visual function[3], [4]. Mouse rod photoreceptor precursor cell grafts can integrate into the host retina and contribute to visual function in both wild-type and degenerate retinae[5]. However, these studies found the benefit to be short-term due to graft loss. Human embryonic stem cell-derived RPE grafts have been shown to rescue visual function in rat and mouse models of retinal degeneration[6]. Although some long-term functional rescue was evident in this study, the rescue steadily decreased with time post-transplantation.

Different mechanisms at each stage of the transplantation procedure may influence graft survival including sub-optimal culture conditions. Subretinal grafts are commonly harvested from cell cultures and transplanted as highly concentrated cell suspensions. In the early post-operative period, graft loss may occur through apoptosis, a particular concern for RPE cell replacement strategies (primary[7], extended life[3], [8]–[14], or stem cell-derived[6]) as these are anchorage-dependent cells and undergo anoikis when dissociated from their basement membrane[15]. Furthermore, grafts are prone to detection by the innate immune system and subsequent attack by the adaptive immune system. Ultimately, transplanted cells that have survived these hazards must also integrate into the host tissue and function as a replacement for the degenerate or dysfunctional host cells. RPE grafts must integrate onto Bruch's membrane and develop apical-basolateral polarity to function[16]. Thus, it is crucial for the transplant to be optimized in terms of preparation, delivery, survival and function.

The present study focuses on the key early stage of graft preparation using the mouse subretinal RPE allograft model. This involves optimizing RPE culture conditions at the crucial stage between harvesting the graft and delivering it to the graft site. Two potential mechanisms of cell death in the prepared graft are apoptosis and necrosis. Apoptosis is a process of programmed cell death and may be initiated from within a cell (intrinsic pathway) or from extracellular ligands (extrinsic pathway). Caspases are proteases of the apoptotic cascade and converge on effector caspases such as caspase-3 and -7, which execute cell death[17], [18]. Membrane disruption such as phosphatidylserine externalization also occurs during apoptosis[19] and can be identified by annexin V binding[20]. Phagocytes detect and clear apoptotic cells via the presence of this phospholipid[21], [22]. Necrotic cells are liable to initiate and propagate an inflammatory response via the release of damage-associated molecular patterns (DAMPs)[23]–[25] and high mobility group box 1 (HMGB1)[26]–[29], which will adversely affect graft survival via activation of the innate immune system.

This study aimed to (a) optimize graft cell preparation by defining the culture conditions which would result in a graft of high viability, and (b) to establish whether the stress of preparing the graft cell suspension adversely affected graft cell viability in vitro and in vivo. Both of these aims were achieved in this study. The data presented here demonstrates that pre-confluent cells should be used to maximize graft cell viability and that the graft preparation procedure used does not adversely affect viability.

## Methods

### Ethics statement

This study was performed in accordance with the ARVO Statement for the Use of Animals in Ophthalmic and Vision Research. Ethical approval for this research was obtained from University College Dublin animal research ethics committee (AREC-P-07-09-Keegan). All surgery was performed under ketamine and medetomidine anaesthesia, and all efforts were made to minimize suffering. C57BL/6 mice were obtained from Harlan UK (Bicester, UK).

### Cell line derivation

The DH01 RPE line was prepared from RPE cultured from a healthy C57BL/10.RIII-H-2^r^ mouse and immortalized using supernatant from the SVU 19.5 cell line[30] secreting retrovirus encoding a temperature-sensitive non-SV40 origin-binding U19 mutant of the SV40 large T antigen (SV40T) and the neomycin resistance gene[31]. Cells were selected in medium containing 0.5 mg/ml Geneticin (G418 Life Technologies, Paisley, UK) for 1–2 weeks. Cells were cloned by limiting dilution in a 96 well plate with progressive 1∶10 dilutions, which were then plated in wells with 250 µl medium and observed daily. When one single cell was present in a well, this was highlighted and allowed to divide. When this culture reached confluence, it was trypsinised and put in a flask to expand up. Cells were shown to express SV40T by immunocytochemistry on 4% formaldehyde-fixed, 0.2% Triton-X-permeabilized cells using a SV40T-specific monoclonal antibody (gift from Dr. P. Jat, Ludwig Institute, London, U.K).

### Cell culture

DH01 cells were routinely cultured in high glucose Dulbecco's modified Eagle's medium (DMEM) (Sigma-Aldrich Ireland, Wicklow, Ireland) supplemented with 1% fetal calf serum (Sigma, Ireland) 200 mM L-glutamine (Sigma, Ireland) and 5 mg/ml penicillin/streptomycin (Sigma, Ireland). As these cells harbour SV40T they were grown at 33°C in 5% CO_2_.

### Appearance of DH01 cells in culture and cell growth assay

DH01 cells were seeded in T75 tissue culture flasks at increasing cell numbers (0.5×10^6^ to 20×10^6^) and cultured for 7 days in full medium. Confluence was defined as the absence of spaces between cells in culture when viewed under phase contrast microscopy. The appearance and status of confluence on Day 7 was captured under phase contrast microscopy using a Nikon TS100 inverted microscope and a Nikon Coolpix 995 digital camera. Cells were counted in triplicate using a haemocytometer. Results are representative of 3 independent experiments.

### Transplant conditions (TC) and baseline conditions (BC)

To determine if the process of preparing a graft cell suspension adversely affected viability, we examined DH01 cells immediately following resuspension in full media (Baseline Conditions, BC) and after being prepared for subretinal transplantation (Transplant Conditions, TC). TC cells were prepared as a highly concentrated cell suspension (50,000 cells/µl), deprived of serum (suspended in serum-free medium), and kept on ice for the maximum period that typically exists between harvesting and transplanting the graft (4 hours).

### Cell viability assay (trypan blue exclusion)

To determine if either confluence or TC affected DH01 cell viability, T75 tissue culture flasks were seeded with 0.5×10^6^ cells/flask (to yield pre-confluent cultures) and 20×10^6^ cells/flask (to yield post-confluent cultures) and cultured for 7 days in full medium. Cell viability was assessed under both BC and TC by counting the percentage of cells that excluded trypan blue. Results represent 3 independent experiments and are presented as mean±standard deviation (SD).

### SDS-polyacrylamide gel electrophoresis

T75 flasks were seeded with 0.5×10^6^ cells/flask (to yield pre-confluent cultures) and 20×10^6^ cells/flask (to yield post-confluent cultures) and cultured for 7 days in full medium. Western blotting was then performed as previously described[32]. 30 µg of total protein was electrophoresed on 12% polyacrylamide gels followed by transfer to nitrocellulose membrane (Schleicher and Schuell, Dassel, Germany) and incubated overnight at 4°C with the antibodies for caspase 3 (Cell Signaling 9662) and caspase 7 (Cell Signaling 9492). An antibody for β-actin (Cell Signaling 4967) was used to confirm equal protein loading between samples. Membrane development was achieved by enhanced chemiluminescence (Amersham, Buckinghamshire, UK). Results are representative of 3 independent experiments.

### Flow cytometry

DH01 cells were cultured to increasing levels of confluence as described in cell growth assay above and examined either under BC or following TC. Cell viability (10^6^ cells per assay) was assessed using a TACS® annexin V (AnV) fluorescein isothiocyanate (FITC) kit (Catalog# 4830-01-K) with propidium iodide (PI) counterstaining following the manufacturer's protocol. Samples were processed by flow cytometry within one hour on a Beckman Coulter CyAn ADP flow cytometer and analysed using FlowJo software. Cells re-suspended in binding buffer alone, and cells stained with AnV only, or PI only, were used to calibrate the machine, control for compensation, and establish regions and gates. Samples were gated to exclude debris, and 20,000 cells were analysed per sample. Results are representative of 4 independent experiments and are presented as mean±SD.

### Subretinal RPE cell transplantation

DH01 cell suspensions (50,000 cells/µl DMEM) were prepared from both pre-confluent and post-confluent cultures as described under Transplant Conditions above. Subretinal transplants were delivered via trans-scleral injection through a glass micropipette to 6 eyes of 6 different C57BL/6 mice. Eyes received either 2 µl of the pre-confluent graft cell suspension (n = 3) or the post-confluent graft cell suspension (n = 3). The animals were euthanized 3 hours post-operatively, the eyes removed and fixed in 4% paraformaldehyde. This early time point was chosen to minimize the risk of post-operative graft cell death due to factors such as anoikis and the host immune response influencing the result observed. The tissue was then cryoprotected in sucrose, embedded in OCT under liquid nitrogen, and stored at −80°C. 7 µm sections were cut on a Leica cryostat and stained as described below.

### Graft Identification (SV40T), Terminal deoxynucleotidyl transferase dUTP nick end labeling (TUNEL) and cleaved caspase 3 immunolabaling of subretinal RPE transplants

Cryosections were stained with 4′,6-diamidino-2-phenylindole (DAPI) to label all nuclei. Graft cells were identified using a specific primary antibody to SV40T (Santa Cruz, SC-20800) and anti-rabbit Texas Red-labeled secondary antibody (Jackson, 111-075-003). Sections were also stained to detect either DNA strand breaks or cleavage of caspase 3. DNA strand breaks were detected by TUNEL as previously described[32]. Cleaved caspase 3 was immunolabeled with a specific primary antibody conjugated to Alexa Fluor 488 (Cell Signaling, #9669S). Sections were mounted using Vectashield hardset (Vector laboratories). Immunostaining was visualized using an Olympus FV1000 confocal microscope. Z-stack images through 3 sections per eye were taken with the microscope settings kept constant for voltage, gain, and offset throughout the series of imaging. Differential interference contrast microscopy (DIC) images were taken at the time of fluorescent confocal microscopy to more accurately identify the subretinal space. The percentage of graft cells (DAPI^+^/SV40T^+^) in the subretinal space that were TUNEL-positive or cleaved caspase 3-positive was calculated and averaged across 3 sections per eye. The results presented are mean±SD.

### Statistical analysis

Data are given as mean±SD. For the in vitro portion of the study, a Student's t-test assuming unequal variances was used for comparisons between groups to determine whether there was a significant difference between the two sample means. Due to the smaller sample sizes for the in vivo analysis, differences in proportions between pre-confluent cell transplants versus post-confluent cell transplants were established by Pearson's Chi-Square test. *Significant at p<0.05; **Significant at p<0.01; ***Significant at p<0.001.

## Results

### DH01 RPE cells are not contact inhibited in culture

**Figure 1:**
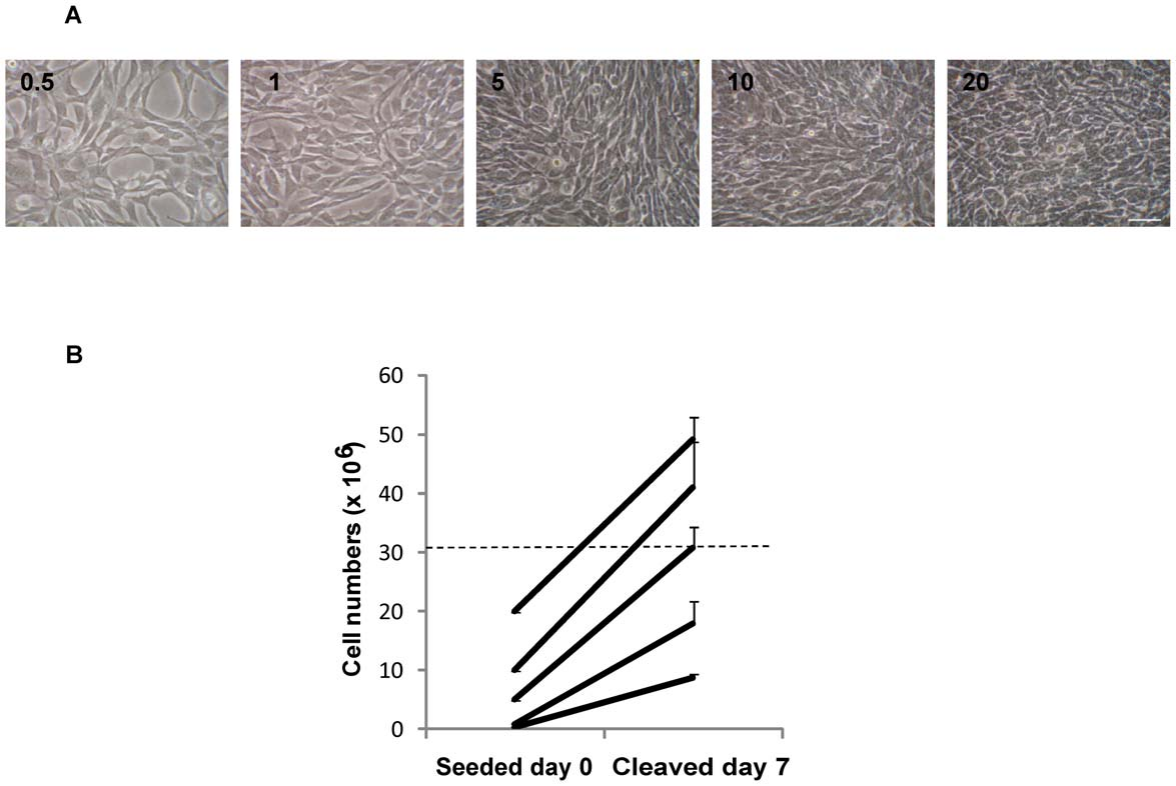
Analysis of cell growth rates demonstrates that DH01 cells are not contact-inhibited. DH01 RPE cells were seeded (Day 0) at increasing cell numbers. Phase contrast images and cell counts were performed on Day 7. (**A**) Phase contrast microscopy of the corresponding appearance on Day 7 of cells seeded on Day 0 (0.5×10^6^ to 20×10^6^) showing increasing cell density. Scale bar equals 50 µm. (**B**). Cell counts on Day 7 of T75 flasks seeded with increasing cell numbers on Day 0. Dotted line approximates cell numbers at confluence. Results are representative of 3 independent experiments.

To determine the cell growth characteristics of DH01 cells, T75 flasks were seeded (Day 0) with increasing numbers of cells (0.5×10^6^ to 20×10^6^ cells/flask) and cultured for 7 days. The corresponding appearance of cultures on Day 7 showed increasing cell density under phase contrast microscopy (Figure 1a) and an increase in cell numbers to pre- and post-confluent levels (Figure 1b). Flasks seeded with 0.5×10^6^ and 1×10^6^ cells were pre-confluent at Day 7. Flasks seeded with 5×10^6^ cells were confluent on Day 7 with 30.9×10^6^±3.5×10^6^ cells. However, DH01 cells were not contact inhibited as higher seeding levels resulted in cell numbers on Day 7 higher than those seen at confluence. 10×10^6^ cells seeded produced 41.2×10^6^±7.6×10^6^ cells, while 20×10^6^ cells seeded resulted in 49.2×10^6^±3.9×10^6^ cells on Day 7. T75 flasks seeded with 10×10^6^ or 20×10^6^ cells were deemed to result in post-confluent cultures.

### Graft cell viability is reduced by culturing DH01 cells post-confluence, but is unaffected by transplant conditions

**Figure 2:**
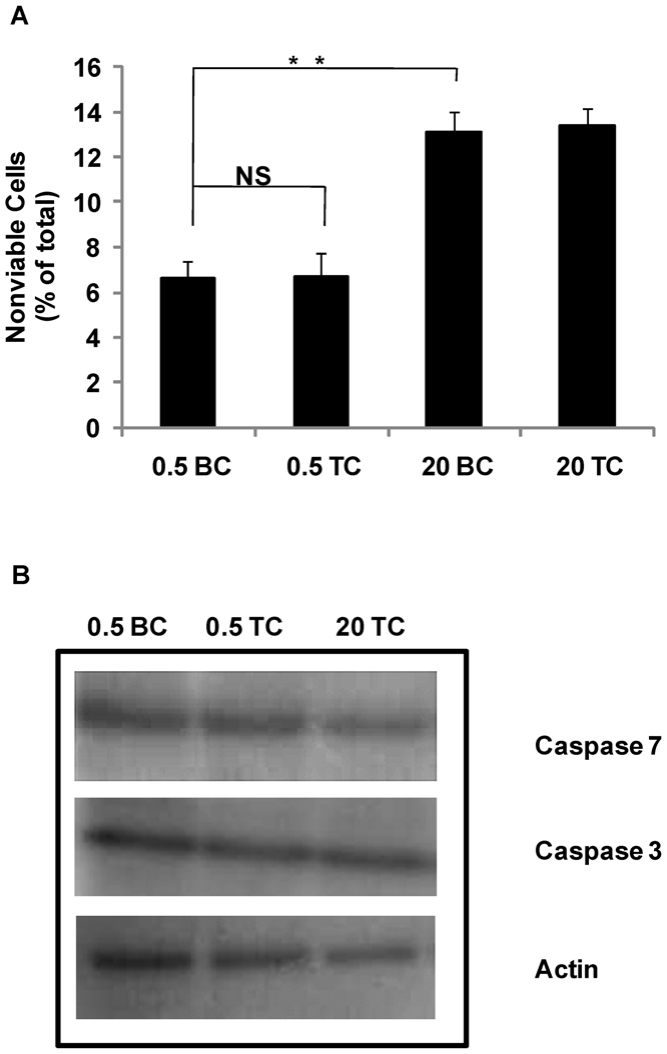
Preparation of DH01 cells under transplant conditions does not induce cell death. (**A**) When 20×10^6^ cells (20) were seeded they exhibited significantly (p<0.01) higher levels of nonviable cells than flasks seeded with 0.5×10^6^ cells (0.5) as measured by trypan blue exclusion, however, transplant conditions (TC) did not significantly affect viability compared to baseline conditions (BC) (p = 0.799). Data are given as mean±SD and are representative of 3 independent experiments. (**B**) SDS-PAGE analysis of caspase 3 and 7 expression in cells seeded at varying cell densities (0.5×10^6^ cells and 20×10^6^) and prepared under baseline and transplant conditions using antibodies sensitive for the pro- and cleaved forms. There was no change in the expression of either caspase 3 or 7 (pro-form) and we did not detect the cleaved fragments. Actin is used as a loading control and results are representative of 3 independent experiments. NS = not significant. **Significant at p<0.01.

We sought to ascertain if culturing DH01 cells post-confluence or the process of preparing cells for transplantation (TC) induced cell death, and more specifically apoptosis. We cultured DH01 cells to pre-confluent (0.5×10^6^ cells seeded) and post-confluent levels (20 x10^6^ cells seeded) and examined on Day 7 for viability (trypan blue exclusion) and caspase activation under BC and TC. Pre- confluent cultures had only background levels of cell death present (0.5 BC: 6.6%±0.8%) that did not increase significantly (p = 0.799) following TC (0.5 TC: 6.7%±1.1%) (Fig. 2a). Levels of cell death in post-confluent cultures increased significantly (p = 0.003) under BC (20 BC: 13.1%±0.9%) compared to pre-confluent cells. However, there was no further increase in cell death following TC (20 TC: 13.4%±0.8%). We then investigated the possible activation of apoptotic pathways by examining the expression of caspases 3 and 7. Figure 2b shows that no difference was detectible in expression of either caspase following TC or in post-confluent cultures. It should also be noted that the antibodies used are for the detection of both the pro- and cleaved forms of caspases 3 and 7, however the cleavage products were not detected.

### Culture of DH01 cells to post-confluence induces both apoptosis and necrosis

**Figure 3:**
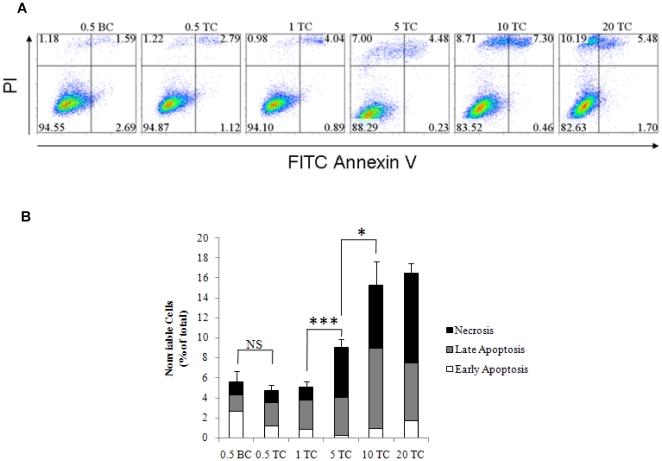
Culture of DH01 cells to post-confluence prior to transplantation induces apoptosis and necrosis. (**A**) Representative flow cytometry dot plots for annexin V (AnV) and propidium iodide (PI) showing that transplant conditions (TC) did not affect cell viability compared to baseline conditions (BC). However, overall levels of late apoptosis (AnV^+^, PI^+^) and necrosis (AnV^-^, PI^+^) increased in cell suspensions prepared post-confluence. (**B**) Graphical representation of the levels of nonviable cells presented as early apoptotic (AnV^+^/PI^-^, white), late apoptotic (AnV^+^/PI^+^, grey) and necrotic (AnV^-^/PI^+^, black). Error bars reflect standard deviation of the total (0.5–20 represent numbers of cells seeded x10^6^ on Day 0; BC = baseline conditions; TC = transplant conditions; NS = not significant; *Significant at p<0.05; ***Significant at p<0.001).

T75 flasks were seeded with 0.5×10^6^ to 20×10^6^ cells, cultured for 7 days yielding pre- and post-confluent cultures (as per Fig. 1b) and then examined under BC or TC. We measured cell death by double-labeling cells with FITC-conjugated AnV and PI (Fig. 3a). Disorganization of the plasma membrane occurs early in apoptosis and is characterized by phosphatidylserine flipping from the inner to the outer leaflet of the plasma membrane. In the presence of Ca^2+^, annexin V binds phosphatidylserine with high affinity, allowing the detection of the apoptotic population by flow cytometry. To differentiate early apoptotic from late apoptotic cells, cells were also stained with PI, a fluorescent indicator of plasma membrane integrity. Annexin V-positive but PI-negative events represent cells in the early stages of apoptosis, whereas events positive for both markers correspond to cells in late apoptosis, when plasma membrane permeability has been compromized. PI-positive but AnV-negative events represent dead cells distinct from apoptosis and were designated necrotic. Analysis (Fig. 3) showed that pre-confluent cells (0.5×10^6^ cells seeded) examined under BC (0.5 BC) had low levels of cell death (0.5 BC: 5.6%±1.1%) that did not increase significantly following TC (0.5 TC: 4.8%±0.5%) (p = 0.218). This population included low levels of both apoptotic and necrotic cells. Flasks seeded at higher levels (1×10^6^ cells), but still pre-confluent on day 7, had similarly low levels of cell death (1 TC: 5.1%±0.5%). However, flasks seeded with 5×10^6^ cells were confluent on day 7 and had significantly higher (p<0.001) levels of cell death following TC (5 TC: 9.0%±0.8%). Cells cultured to post-confluence showed a progressive increase in overall levels of cell death with the maximum levels seen in cultures seeded with 20×10^6^ cells (20 TC: 16.5%±0.9%). This population was predominantly accounted for by necrotic cells (AnV^-^/PI^+^: 9.0%±1.2%) and cells in the late stages of apoptosis (AnV^+^/PI^+^: 5.8%±1.0%). A summary of these results is displayed in Table 1.

**Table 1:** Summary of rates of cell death examined under increasing levels of cell confluence (0.5 to 20) and under baseline conditions (BC) or transplant conditions (TC).

	Early Apoptosis (%)	Late Apoptosis (%)	Necrosis (%)	Total Nonviable (%)
	AnV^+^/PI^-^	AnV^+^/PI^+^	AnV^-^/PI^+^	AnV^+^/PI^-^ & AnV^+^/PI^+^ & AnV^-^/PI^+^
**0.5 BC**	2.6±0.2	1.7±0.5	1.3±0.8	5.6±1.1
**0.5 TC**	1.2±0.1	2.3±0.2	1.3±0.7	4.8±0.5
**1 TC**	0.8±0.1	3.0±0.6	1.3±0.3	5.1±0.5
**5 TC**	0.2±0.1	3.8±0.5	5.0±1.0	9.0±0.8
**10 TC**	0.9±0.3	8.0±0.7	6.3±2.2	15.2±2.4
**20 TC**	1.8±0.5	5.5±1.0	9.0±1.2	16.5±0.9

Flow cytometric analysis of cell suspensions prepared from increasingly confluent cultures examined either immediately following resuspension (baseline conditions, BC) or after undergoing the transplant preparation procedure (transplant conditions, TC). Flasks seeded with 0.5×10^6^ (0.5) and 1×10^6^ (1) cells were pre-confluent at Day 7. Flasks seeded with 5×10^6^ cells (5) were confluent on Day 7. Flasks seeded with 10×10^6^ cells (10) and 20×10^6^ cells (20) resulted in increasingly post-confluent cultures. Cells were stained with annexin V (AnV) and propidium iodide (PI). The data demonstrates that cells from pre-confluent cultures have low levels of total cell death (0.5 BC: 5.6%±1.1%) and that this does not increase following TC (0.5 TC: 4.8%±0.5%). However, cells from post-confluent cultures have progressively increasing levels of cell death (up to 20 TC: 16.5%±0.9%).

### Transplanting pre-confluent cells increases graft cell viability in an in vivo model

**Figure 4:**
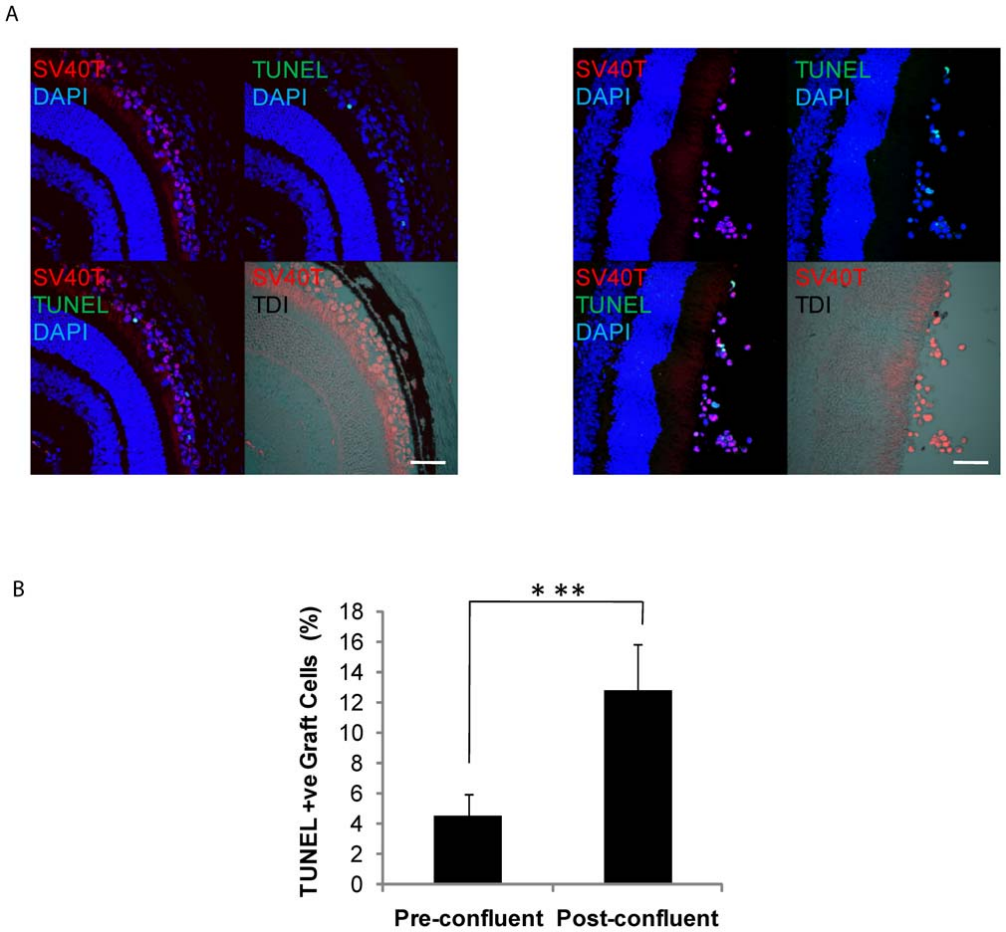
Pre-confluent grafts have greater viability in vivo. (**A**) DH01 allografts 3 hours post-transplant. Graft cells are identified via immunolabeling SV40T (Texas red) and apoptotic/necrotic cells via TUNEL-labeling (FITC, green). All nuclei are stained with DAPI (blue). The subretinal location of the graft cells is highlighted in the DIC images. Levels of cell death were lower in grafts of pre-confluent cells **(left)** than post-confluent cells **(right)**. Scale bar 50 µm. (**B**) There is a significant increase in levels of cell death in grafts of post-confluent cells compared to grafts prepared from pre-confluent cultures. ***Significant at p<0.001.

**Figure 5:**
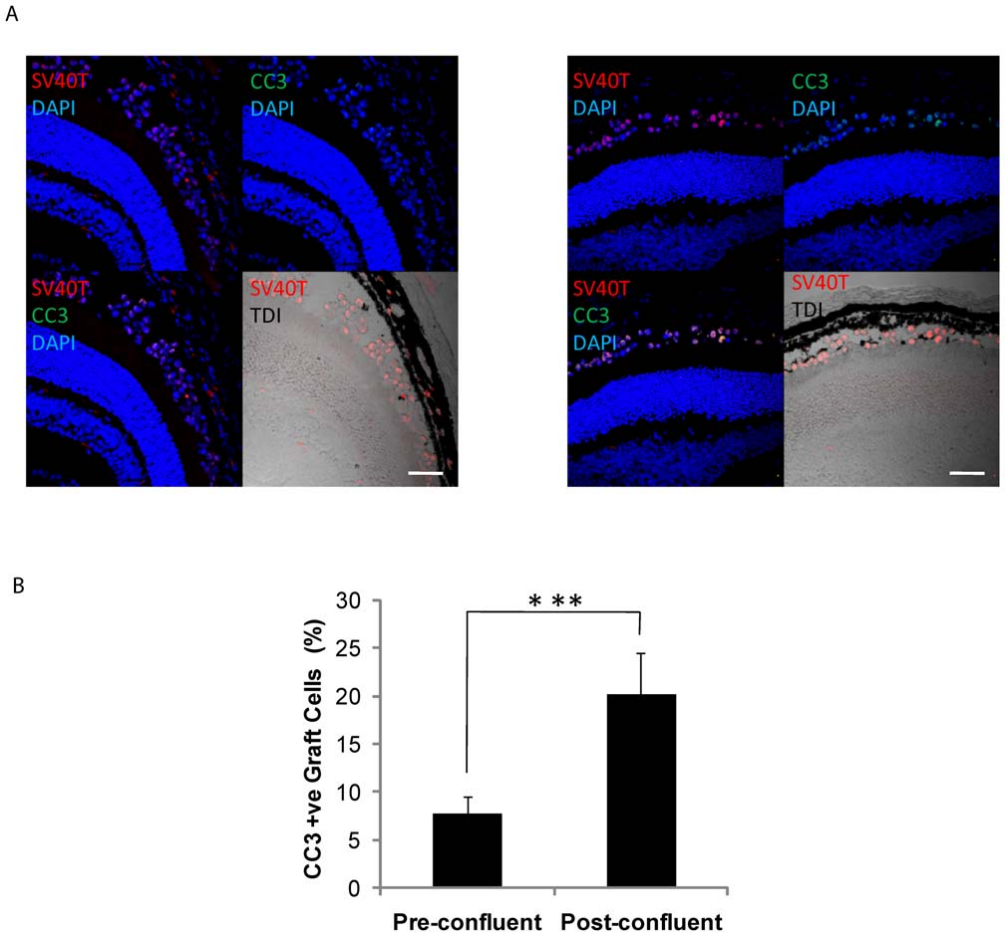
Pre-confluent grafts have less apoptosis in vivo. (**A**) DH01 allografts 3 hours post-transplant. Graft cells are identified via immunolabeling SV40T (Texas red) and apoptotic cells via cleaved caspase 3 (CC3) immunolabeling (Alexa Fluor 488, green). All nuclei are stained with DAPI (blue). The subretinal location of the graft cells is highlighted in the DIC images. Rates of cleaved caspase 3 were lower in grafts of pre-confluent cells **(left)** than post-confluent cells **(right)**. Scale bar 50 µm. (**B**) There is a significant increase in rates of apoptosis in grafts of post-confluent cells compared to grafts prepared from pre-confluent cultures. ***Significant at p<0.001.

Pre-confluent and post-confluent DH01 cells were prepared under TC and transplanted to the subretinal space of allogeneic mice (n = 3 eyes in each group). Eyes were removed 3 hours post-operatively and tissue sections cut. Graft cells were identified by SV40T immunolabeling. Apoptotic and necrotic cells were detected by TUNEL-labeling, a method for detecting DNA fragmentation by labeling the terminal end of nucleic acids. Subretinal allografts of DH01 cells prepared from pre-confluent cultures had lower levels of TUNEL-positive cells compared to grafts of post-confluent cells (Fig. 4a). Three sections from each graft were imaged and the average percentage of TUNEL-positive graft cells in the subretinal space calculated. This demonstrated a significant increase (p<0.001) in the percentage of TUNEL-positive cells following transplantation of post-confluent cells (12.8%±3.1%) compared to pre-confluent cells (4.5%±1.4%) (Fig 4b). To validate this result, three sections per eye were also immunolabeled for cleaved caspase 3 (Fig. 5a) and analyzed as per the TUNEL staining described above. This confirmed significantly greater (p<0.001) levels of caspase 3 cleavage in post-confluent grafts (20.2%±4.3%) compared to pre-confluent grafts (7.8%±1.8%) (Fig. 5b).

## Discussion

Subretinal cell transplants are commonly harvested from cell cultures and delivered as high concentration cell suspensions. In vitro and in vivo these cells are exposed to local conditions that are likely to influence cell growth and performance. Primary cell lines are troublesome due to limited access, complex harvesting techniques, and unstable phenotype after few passages in culture. Transformed cell lines are therefore an attractive option for ease of access and reproducibility of genotype and phenotype within and between laboratories. This study used the mouse RPE cell line, DH01, to optimize graft preparation and survival. We found that DH01 is not contact inhibited in culture as cell numbers continued to increase even after cultures reached confluence. Pre-confluent DH01 cells in suspension had 6.6% nonviable cells, but this approximately doubled to 13.1% using post-confluent cultures. Although it appears optimal to culture high numbers of cells to ensure an adequate graft is available for transplantation, our findings refute this approach. Although more cells will be available in post-confluent cultures, we have shown that a higher proportion of these will be nonviable.

Adverse effects of the graft preparation procedures on cell viability should be minimized. One important difference between cell transplants and solid tissue or organ transplants is that cell transplants are often derived from cell cultures and delivered as a cell suspension (stem cells[33], Schwann cells[34], [35], pancreatic islet cells[36] and RPE cells[2]-[4]). We found that pre-confluent cultures had significantly lower levels of nonviable cells than post-confluent cultures. However, the graft preparation procedure (TC) did not adversely affect viability in grafts prepared from pre-confluent or from post-confluent cultures (Figs. 2a and 3, Table 1).

We investigated whether cell death in post-confluent cultures was due to apoptosis but consistently found no difference in caspase activation between pre-confluent cultures (BC and TC) and post-confluent cultures (TC) (Fig. 2b). We did find that levels of early apoptosis remained low (1–2%) in pre- and post-confluent cultures, but the proportion of cells in the late stages of apoptosis increased from just 2% in pre-confluent cultures to between 6% and 8% in post-confluent cultures. However, the biggest change in cell death was due to necrosis, increasing from 1% in pre-confluent cultures to 9% in post-confluent cultures. The predominance of necrosis over apoptosis in graft cell loss has been previously observed in Schwann cell transplantation to the spinal cord where six times more cells died by necrosis than by apoptosis during the first week after transplantation[34]. We also confirmed that pre-confluent cultures were 95% viable and this did not decrease following the graft preparation procedure (Figs. 2 and 3).

Finally, we wanted to confirm that pre-confluent cultures resulted in greater graft viability than post-confluent cultures in vivo. To achieve this, we employed a mouse model of subretinal transplantation and examined subretinal grafts for cell death three hours post-operatively by TUNEL-labeling. This early post-operative time-point was chosen in order to minimize the risk of post-operative graft cell death due to factors such as anoikis and the host immune response influencing the result observed in this study which aimed to optimize graft preparation. DNA fragmentation is not exclusive to apoptosis. Necrosis can also be accompanied by DNA breaks and thus TUNEL-labeling also detects necrotic cells[37]-[39]. The DH01 cell line is immortalized using the nuclear protein SV40T. Therefore, we were able to use this marker in conjunction with TUNEL-labeling to determine the rates of apoptosis/necrosis in our subretinal transplants. We found that grafts from pre-confluent cultures had a lower proportion of TUNEL-positive cells than grafts from post-confluent cultures (Fig. 4a). This difference is consistent across a series of transplants (Fig. 4b) and closely mirrors our in vitro data (Figs. 2a and 3b). Caspase 3 is a key executioner of apoptosis and is activated when cleaved by upstream signals[40]. To validate the data seen with TUNEL-labeling, sections were also immunolabeled for cleaved caspase 3. This confirmed greater rates of caspase 3 cleavage in post-confluent grafts compared to pre-confluent grafts. Rates of caspase 3 cleavage were higher than rates of TUNEL-labeling. This may be due to the identification of cells in earlier stages of apoptosis that have not yet developed DNA fragmentation demonstrable by TUNEL-labeling. Another method that could be considered to compare graft viability in vivo between the two groups is PI co-administration with the graft cell suspension[41], [42]. Thus, the in vivo data was consistent with the in vitro data and found that using pre-confluent cells resulted in significantly lower levels of graft apoptosis/necrosis in the subretinal space three hours post-operatively.

High levels of necrosis and apoptosis are likely to impact on graft rejection as well as graft performance. Necrosis is an inherently more inflammatory process than apoptosis and causes an inflammatory response via the release of molecules such as DAMPs[23]–[25] and HMGB1[26], which can stimulate monocytes and macrophages[25], [27]–[29], [43], enhance dendritic cell maturation[44]–[46], and promote natural killer cell chemotaxis and cytolytic activity[47]. Phagocytes can also detect and clear apoptotic cells via the presence of externalized phosphatidylserine[21], [22]. Although apoptotic cells do not release their HMGB1[26], when engulfed by macrophages apoptotic cells actually induce the phagocytes to release HMGB1[48]. Thus, although low grade apoptosis does not induce inflammation, extensive apoptosis is capable of inducing an inflammatory response[49]. High levels of apoptosis and necrosis in the graft are therefore likely to activate local innate inflammatory immune responses, some of which may target the graft.

Graft necrosis and apoptosis, together with activated local inflammation, also increase the risk of an allogeneic adaptive immune response. Necrotic debris can be engulfed by phagocytes which may act as antigen-presenting cells and trigger an adaptive immune response against the graft[50]. Extensive apoptosis can trigger dendritic cell maturation, antigen-presentation and an adaptive immune response[51]–[53]. Consequently the remaining cells within the transplant, although neither apoptotic nor necrotic, could be presenting specific antigens to clonally expanding T- and B-cells primed to attack these allogeneic cells. Therefore it is critical to minimize apoptosis and necrosis in the graft in order to minimize the risk of graft failure.

It follows therefore that the benefits of minimizing apoptosis and necrosis within a graft are threefold: apoptosis and necrosis are themselves inherent mechanisms of graft cell loss and graft failure; these nonviable cells are likely to trigger a local inflammatory innate immune response at the graft site; and finally, perhaps most significantly, the more of these dead cells that are cleared by phagocytes, the higher the risk of consequent targeted graft attack by the adaptive immune system. These issues are now the subject of further in vivo studies and investigation.

Transplantation of cell suspensions to the subretinal space is now being superseded by the transplantation of patches, i.e. of cells on the platform on which they were cultured. There are several good reasons for this. For example, graft cell suspensions when transplanted to the subretinal space have an unpredictable distribution due to the fluid nature of the graft. In contrast, transplantation of a sheet of graft cells cultured on a platform offers the possibility of controlling graft size and position. For RPE grafts in particular, anoikis[15], is a potential source of graft failure in RPE cell suspension transplantation that may be overcome by transplantation on a platform. A crucial function of RPE cells is maintenance of the outer blood-retinal barrier. To achieve this, it is vital that these cells are confluent and create tight junctions to prevent the free passage of water and large molecules from the choriocapillaris to the subretinal space. For this reason it will be desirable in the future to culture RPE cells to confluence on a platform prior to transplantation. However, our findings demonstrate that care must also be taken not to culture the cells beyond confluence due to rising levels of apoptosis and necrosis and the problems this may provoke in terms of initiating an inflammatory or immune response against the graft.

There is currently no permanent treatment for retinal degenerations. However transplantation offers an attractive possibility. The success of this approach will require optimum graft survival and function; however no previous work has described best practice for graft cell preparation as illustrated in this paper. Our future work will examine the use of patch transplants to enhance graft survival and on modification of the innate immune response. The future success of subretinal transplantation will be determined by the ability to prepare grafts to a reproducibly high standard, with maximum graft viability and function, in conjunction with minimum inflammatory and immune reactions.
